# Circulating *Brucella* species in wild animals of the Serengeti ecosystem, Tanzania

**DOI:** 10.1186/s42522-021-00047-6

**Published:** 2021-08-24

**Authors:** R. M. Sambu, C. Mathew, H. E. Nonga, A. S. Lukambagire, R. B. Yapi, J. Akoko, G. Fokou, J. D. Keyyu, B. Bonfoh, R. R. Kazwala

**Affiliations:** 1 Ministry of Livestock and Fisheries, P.O. Box 129, Mwanza, Tanzania; 2 College of Veterinary Medicine and Biomedical Sciences, Sokoine University of Agriculture, P. O. Box 3021, Chuo Kikuu, Morogoro, Tanzania; 3 Ministry of Livestock and Fisheries, P.O. Box 2870, Dodoma, Tanzania; 4 Centre Suisse de Recherches Scientifiques en Côte d’Ivoire, Abidjan, Côte d’Ivoire; 5 Université Alassane Ouattara, Bouaké, Côte d’Ivoire; 6 Department of Biomedical Sciences and Technology, Maseno University, Kisumu, Kenya; 7 Tanzania Wildlife Research Institute, Box 661, Arusha, Sambu, Tanzania

**Keywords:** Brucellosis, Serengeti ecosystem, Wildlife, Zoonosis, One Health

## Abstract

**Background:**

Brucellosis is a bacterial zoonosis of public health and economic importance worldwide. It affects a number of domestic animals, wild animals and humans. Human brucellosis originates from either livestock or wildlife. The species of *Brucella* circulating in wild animals in Tanzania is largely unknown due to insufficient surveillance. This study was carried out to identify *Brucella* species found in selected wildlife hosts in the Serengeti ecosystem.

**Methodology:**

The study used a total of 189 archived samples that were obtained from cross-sectional studies previously conducted between 2000 and 2017 in the Serengeti ecosystem in Tanzania. Whole blood, serum and amniotic fluid collected from buffalos, lions, wildebeest, impala, zebra and hyena were available for DNA extraction. Multiplex polymerase chain reaction for *B. abortus*, *B. melitensis*, *B. ovis* and *B. suis* (AMOS PCR) and quantitative real-time PCR (qPCR) targeting the bcsp31 and IS*711* genes for *Brucella* genus detection and the IS*711* targets *alkB* for *B. abortus* and *BMEI*1162 for *B. melitensis* were used to detect *Brucella* strains.

**Results:**

Out of the 189 samples tested, 12 (6.35 %) and 22 (11.6 %) were positive to AMOS-PCR and qPCR, respectively. Most of the positive samples were from lions (52.6 %) and buffaloes (19.6 %). Other animals that were positive included: wildebeest (13.6 %), impala (13.6 %), zebra (4.5 %) and hyena (4.5 %). Out of 22 positive samples, 16 (66.7 %) were identified as *B. abortus* and the other six samples did not amplify for neither *B. abortus* nor *B. melitensis*.

**Conclusions:**

The detection of *Brucella* DNA in archived wild animal samples shows testing potential of samples collected from this population. The zoonotic species *B. abortus* and *B. melitensis* detected in wild animals have previously been reported in livestock and humans in the region. The findings suggest that, due to the contact network, some of the identified wild animal hosts in this study could be reservoirs for infections in domestic animals and humans within the Serengeti ecosystem while others are likely dead-end hosts. One Health control strategies and continuous surveillance programs in other wildlife reserved areas should be implemented to help predicting transmission in livestock and humans in the region.

**Supplementary Information:**

The online version contains supplementary material available at 10.1186/s42522-021-00047-6.

## Introduction

Brucellosis affects a number of domestic and wild animal species as well as humans [1, 2]. The disease is a public health problem that is challenging to control in many developing countries including Tanzania, especially in pastoral and agro-pastoral farming systems [3–5]. According to the World Health Organization (WHO), brucellosis is an important re-emerging, neglected tropical zoonosis [6] largely due to lack of awareness, and minimal investment in surveillance and control measures.

In wild animals, brucellosis occurs as a result of spill-over from infected livestock or as a natural, sustained infection within susceptible wild animal populations [7, 8]. Wild ungulates can acquire infection by ingesting contaminated forage [8]. Carnivores such as wolves (*Canis lupus*) and foxes (*Vulpes vulpes*) are thought to be exposed through the ingestion of infected animals, placentae or aborted fetuses [2]. The disease has been reported in wild animals in some African countries, including Kenya [9], South Africa [8], Zimbabwe [10] and Tanzania [11–14]. Among Tanzanian wild animals, *Brucella* infections have been reported in topi (*Damaliscus lunatus jimela*), buffalo (*Syncerus caffer*), impala (*Aepyceros melampus*), Thompson gazelle (*Eudorcas thomsonii*) and wildebeest (*Connochaetes*) [15, 16]. However, most of these reports were based on serological studies, without identification of the *Brucella* spp. involved. Other studies reported brucellosis in livestock-wildlife interfaces in the Ngorongoro Conservation Area [17] and the Mikumi-Selous Ecosystem [18].

In recent years, many African countries have prioritized zoonotic diseases under the Global Health Security Agenda and brucellosis has been ranked among priority zoonotic diseases for control [19, 20]. In Tanzania, in particular, brucellosis ranks sixth among the priority zoonoses that the country focuses its control efforts on [16, 21]. Since the prioritization of brucellosis in 2017, a number of efforts for control, including development of a national control strategy, enacted vaccine and vaccination regulation and vaccination campaigns have been put in place. Critically highlighted areas include the contribution of different hosts to the transmission and maintenance of the disease in the country [16]. Studies on brucellosis in Tanzania have shed light on *Brucella* species circulating in the different livestock species within different regions [17, 18, 22]. However, *Brucella* spp. strains circulating in wild animal populations remain under-reported [16, 21]. The aim of this study was to identify the *Brucella* species circulating among wild animals in the Serengeti ecosystem in Tanzania, using molecular techniques and to evaluate the usefulness of archived samples in yielding information on circulating *Brucella* spp. By using clinical/field samples archived for up to 15 years, the study sought to detect and characterize *Brucella* DNA extracted directly from samples, most of which were not viable for bacteriological culture and/ or serological exploration.

## Materials and methods

### Study area

**Fig. 1:**
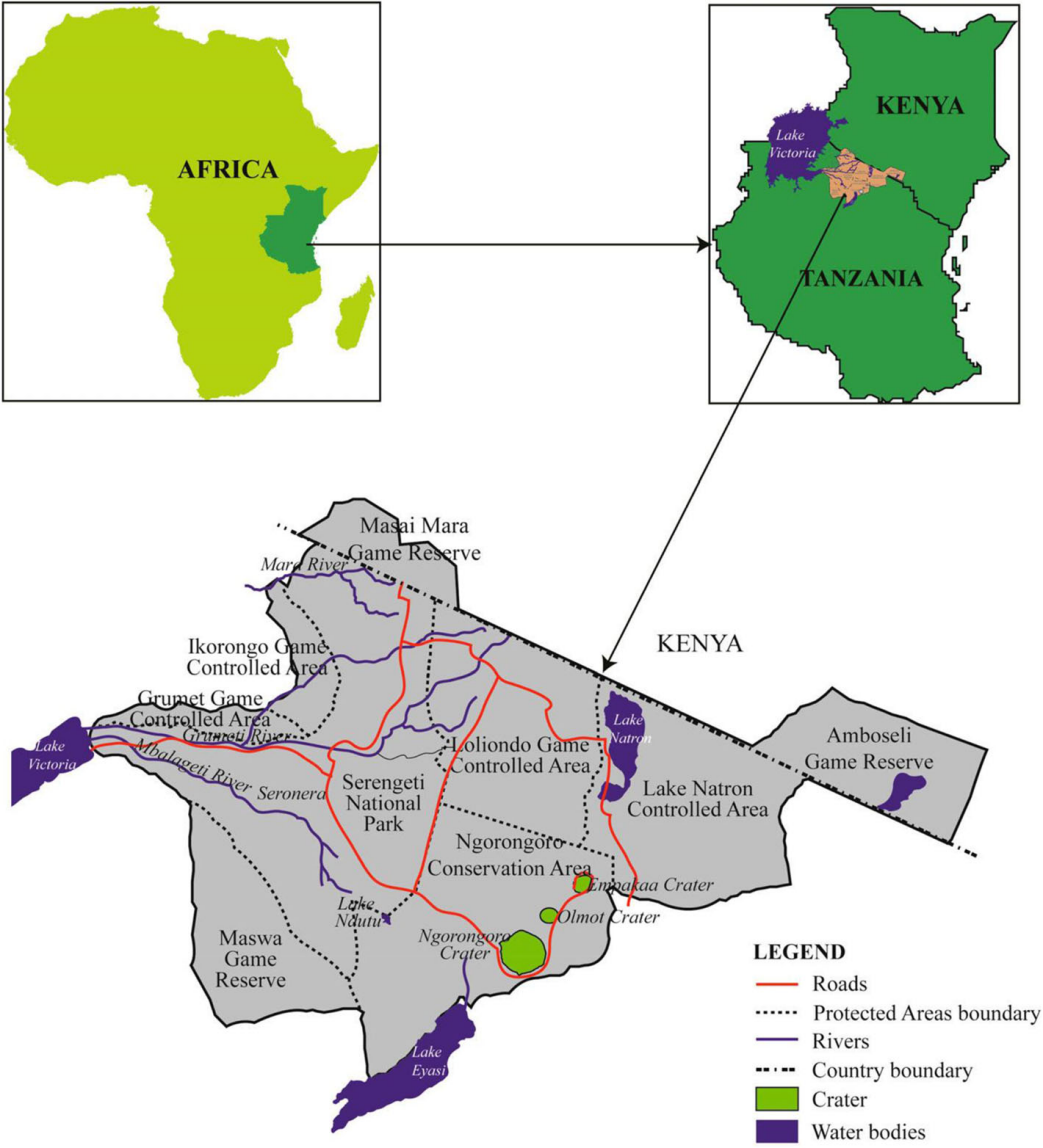
A map of the study area showing the Serengeti ecosystem [26]

The samples used were collected during various cross-sectional studies previously conducted in the Serengeti ecosystem in Tanzania. The Serengeti ecosystem is located in the northwest of the country between the Ngorongoro highlands and Lake Victoria. This ecosystem comprises of Serengeti National Park, the Ngorongoro Conservation Area, Maswa Game Reserve, Loliondo Game Controlled Area and Kenya’s Masai Mara National Reserve (Fig. 1). The study area was selected because there is notable interaction between wild animals, livestock, and humans. The area is mainly inhabited by the Maasai, with livestock keeping as their main socio-economic activity [23]. Furthermore, there have been previous reports on brucellosis in humans and livestock around the ecosystem [22, 24, 25].

### Collection of biological samples

All samples were retrieved from the archive of the Tanzania Wildlife Research Institute (TAWIRI) bio-repository in Arusha and the Serengeti laboratory. Whole blood (collected in the EDTA tubes), serum and amniotic fluid samples were used in the present study. The samples were collected between 2000 and 2017 during routine surveillance, research and veterinary training programs. From TAWIRI and the Serengeti laboratory, all samples were transported in cold chain and stored at -20 °C at the microbiology laboratory, college of Veterinary Medicine and Biomedical Sciences in Sokoine University of Agriculture (SUA) Tanzania. The retrieved samples were originally collected from buffaloes, wildebeest, zebra, lions, baboon, impala and hyena. These were the only samples available for this study. In total, 189 samples were used, out of which 11 were amniotic fluid, 170 whole blood and eight serum samples.

### Molecular detection of *Brucella* spp

The study employed a conventional AMOS PCR for the detection of *B. abortus* biovars 1, 2, and 4, *B. melitensis* biovars 1, 2, and 3, *B. ovis* and *B. suis* biovars 1, 2, 3, 4 and 5. A quantitative real -time PCR (qPCR) for the detection of *Brucella* spp. from DNA extracts was also used. DNA extraction from samples was done at the microbiology laboratory, college of Veterinary Medicine and Biomedical Sciences in SUA Tanzania. A commercial DNA extraction kit (Zymo Research, USA Genomic DNA Tissue Mini Prep kit) was used as previously described [27]. Briefly, 40 µl of genomic lysis buffer were added to 200 µl of the source sample. The mixture was subjected to digestion, deactivation, washing and elution steps as per manufacturer’s instructions. Stock DNA samples were stored at -20 ºC until PCR was performed.

The conventional AMOS PCR was run as previously described [28]. Briefly, a reaction mixture of a final concentration of 0.5µM for each of the primers (forward and reverse), 5 µl of the DNA template and x1 concentration of the OneTaq Quick-Load DNA Polymerase PCR master mix (New England BioLabs, Mass., USA) were prepared up to a final volume of 25 µl. After an initial denaturization step of 5 min at 95 °C in a thermo cycler (TaKaRa, Japan), the mixture underwent 35 cycles of denaturization at 94 °C for 1 min. Annealing at 53 °C for 30 s, extension at 72 °C for 7 min, and final extension steps at 72 °C for 10 min were then performed. Amplification of the target region was confirmed based on the presence of specific bands for the different *Brucella* spp. The PCR products (3 µl) were analyzed on a 1.5 % agarose gel pre-stained with bromide dye (Invitrogen Carlsbad, CA) run at 100 V for 60 min for electrophoresis detection and direct visualization. The primers used in this analysis were obtained from Bioline Inc (Taunton, MA, USA) as previously described [28].

The qPCR for *Brucella* genus identification targeted *bcsp31* and *IS711* gene regions as previously described [29, 30]. All qPCR assays were run on the Premier instruments (Biosoft International, Palo, Alto, Calif.) at a final volume of 25 µl, consisting; 12.5 µl of 2 X master mix, 2.5 µl of purified DNA template, 2.5 µl of internal positive control (IPC) master mix and 0.5 µl of IPC synthetic DNA from the Luna Universal Probe One-Step real-time qPCR premix kit (New England BioLabs, Mass., USA). After an initiation at 50ºC for 2 min and denaturation 95ºC for 10 min, activation of the polymerase enzyme followed by 40 cycles of: 95 ºC for 15 s, and 60 ºC for 1 min thermocycling, repeated for approximately 100 min. Samples were considered positive only if they amplified in both *bcsp31* and *IS711* targets and below a predetermined cycle time (< 39).

Samples positive for the *Brucella* genus level target were then subjected to a multiplex assay to distinguish *B. abortus* from *B. melitensis*. The assay used *B. abortus* and *B. melitensis* primers targeting the insertion sequences downstream of *alkB* and *BMEI1162* targets respectively [30]. Analysis was done according to manufacturer instruction in the *Brucella* genus Genesig standard kit (Genesig Camberly, UK). A volume of 10 µl DNA was mixed with primers and probes in 1000 µl reaction tubes as detailed in Probert et al. [30]. Primers and probes used in the qPCR assay for the detection of *Brucella* spp. are described elsewhere [29]. Amplification and real-time fluorescence detection were performed on the iCycler real-time PCR detection system (Bio-Rad Laboratories, Hercules, Calif.).

The results from each of the techniques were collated in Microsoft Excel then descriptive and analytical statistics were done using R software [31]. A positive result was considered only if a sample was positive by both qPCR assays, or by the AMOS PCR. Proportions of positivity by the qPCR assay were then estimated for each category of variables.

Cross tabulation was used to determine the diagnostic sensitivity and specificity of the AMOS and real-time qPCR using the qPCR speciation assay as the reference test.

## Results

Samples from seven wild animal species, buffaloes, wildebeest, zebra, lions, baboons, impala and hyenas, were used in the present study. Out of 189 identified samples, 170 were whole blood collected in EDTA tubes, eight were sera and 11 amniotic fluid samples. Most of the samples (80; 42.3 %) were from wildebeest, and a larger proportion of the samples (183; 96.8 %) were also obtained from female animals. In terms of specific location, the majority (115; 60.9 %) of samples were from the Serengeti National Park while the rest were from the Ngorongoro conservation area and National Park. It was found that the age of the wild animal sampled (adult), location (Serengeti) and the sample type used for DNA extraction (whole blood) were all significantly associated with the detection of *Brucella* DNA (Table 1).

**Table 1:** Characteristic features of whole blood, serum and amniotic fluid samples from wild animals of the Serengeti Ecosystem (*n* = 189)

Variable	Categories	Number of samples tested (%)	Positive (qPCR)	Percentage positive
Sex	Female	183 (96.8)	20	11.0
	Male	6 (3.2)	2	33.3
Age (group)	Adult	186 (98.4)	20	10.8
	Sub-adult	3 (1.6)	2	66.7
Location	Serengeti	115 (60.9)	15	13.0
	Ngorongoro	74 (39.2)	7	9.5
Species	Buffalo	46 (24.3)	7	15.2
	Wildebeest	80 (42.3)	3	3.8
	Zebra	25 (13.2)	1	4.0
	Lion	19 (10.1)	7	36.8
	Baboon	5 (2.7)	0	0.0
	Impala	10 (5.3)	3	30.0
	Hyena	4 (2.1)	1	25.0
Sample type	Whole blood	170 (90.0)	19	11.1
	Serum	8 (4.2)	1	14.3
	Amniotic fluid	11(5.8)	2	18.2

This data stems from a study conducted between 2000 and 2017 in the Serengeti National Park and Ngorongoro Conservation area

**Fig. 2:**
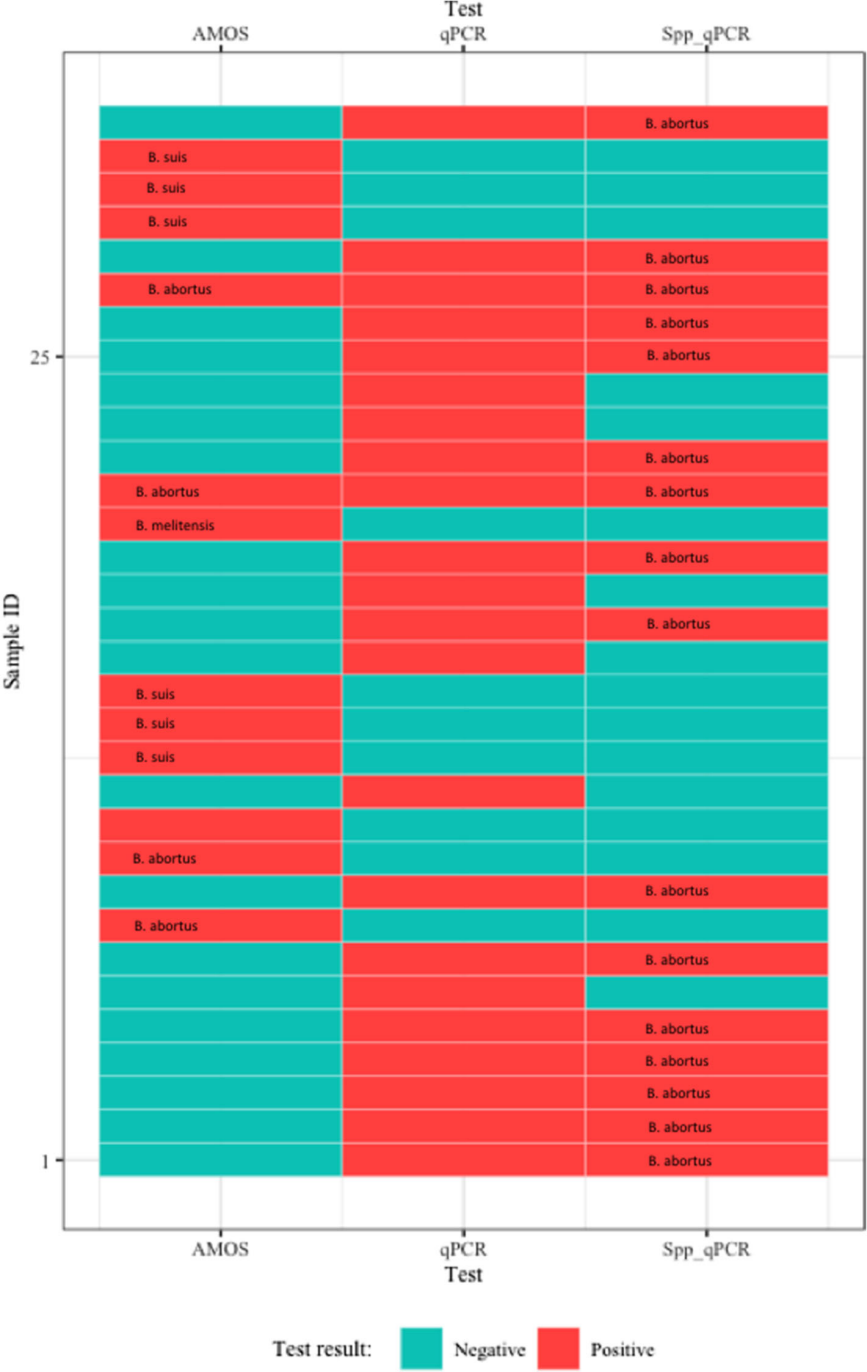
Schematic aggregate plot for the samples tested positive for the three assays (AMOS PCR, qPCR genus specific and species specific), *Brucella* spp. identified and the respective agreement across tests

Of the 189 samples screened, *Brucella* DNA was identified in 12 (6.3 %) samples (nine whole blood, one serum and two amniotic fluid) based on AMOS PCR (Supplementary material S1). Out of the 12 positive samples, *Brucella* species identified included four *B. abortus*, one *B*. *melitensis* and six *B. suis* and one was unidentified (Fig. 2). The animal species distributions of *Brucella* DNA positive samples based on AMOS PCR and the qPCR speciation assay are detailed in Table 2.

**Table 2:** *Brucella* spp. detected by AMOS PCR and qPCR speciation assay in wild animals from the Serengeti ecosystem (*n* = 189)

Wild animal spp.	Tested (n)	Test method	
		**AMOS PCR** (***n*** = 12)	**qPCR speciation** (***n*** = 16)
		***Brucella*spp. (%)**^a^	***Brucella*spp. (%)**^**a**^
Lion	19	*Brucella abortus* (25)	*B. abortus* (38.0)
Buffalo	46	*Brucella abortus* (8.3)	*B. abortus* (43.8)
Wildebeest	80	*Brucella suis* (33.3)	NA
Zebra	25	*Brucella suis* (8.3)	*B. abortus* (6.3)
Impala	10	*Brucella melitensis* (8.3)	*B. abortus* (12.5)
Hyena	4	*Brucella suis* (8.3)	NA
Baboon	5	NA	NA

*NA *No amplification

^a^Positivity proportions calculated by column in all cases

The qPCR test results indicated that 22 samples (11.6 %) were positive for *Brucella* DNA. Overall, 16 samples out of 22 (72.7 %) samples were positive for *B. abortus* in the real-time qPCR speciation assay and six samples did not amplify for either species (Fig. 2). The 16 samples that were positive for *B. abortus* included two samples that were also positive for the same species in AMOS PCR. Using the real-time speciation assay as the reference test, AMOS PCR had a sensitivity of 16.7 % and specificity of 92 %, while the qPCR assay had a sensitivity of 72.7 % and specificity of 100 % (Table 3). The full data set for samples positive by all the tests are shown in the supplementary material (S2).

**Table 3:** Cross tabulation of the molecular tests used, with real-time qPCR speciation assay as the reference

	Real-time Speciation		
	Positive	Negative	Total
**AMOS-PCR**			
Positive	2 (0.2)	10 (0.1)	12 (0.1)
Negative	14 (0.1)	163 (0.9)	173 (0.9)
**Real-time qPCR**			
Positive	16 (0.7)	6 (0.0)	22 (0.1)
Negative	0 (0)	167 (1.0)	167 (0.9)
**Total**	16 (0.1)	173 (0.9)	189 (100)

## Discussion

The purpose of the current study was to determine the *Brucella* species circulating among wild animals in the Serengeti ecosystem in Tanzania, using molecular techniques and to evaluate the usefulness of archived samples yielding information on *Brucella* spp. Zoonotic *Brucella* spp. were detected in wild animal populations in the Serengeti ecosystem using qPCR and AMOS PCR. Lions and buffaloes had the highest proportions of positivity from the sample pool. The most identified species in the wild animals was *Brucella abortus* although *B. melitensis* was also detected. This is the first reported study to conduct molecular detection of *Brucella* directly from archived samples of wild animals from Africa. Detecting *Brucella* circulating in the blood is quite rare and that this detection method underestimates infection rates because *Brucella* is hiding out in other tissues since this is an intracellular parasite. However, other studies have indicated serum as a preferred sample source for *Brucella* detection [32].

Results obtained from qPCR show that *B. abortus* was dominant in the samples collected suggesting that it is a common *Brucella* species circulating in the Serengeti ecosystem. Detection of *Brucella* spp. from the study area is not surprising, as previous studies have reported *Brucella* sero-positivity that ranged between 10.5 and 17 % [14] in wild animals in Tanzania including the Serengeti ecosystem [12, 22]. Therefore, detection of pathogenic DNA in samples collected from wild animals in the study area, further confirms that *Brucella* is circulating in the ecosystem.

It was further observed that *Brucella* DNA were detected more in lions (25 % by AMOS PCR and 38 % by qPCR) or buffaloes (8.3 % by AMOS PCR and 43.8 % by qPCR) than in other wild animal species. It is probably, that this was the case because lions are indiscriminate carnivores and are likely to prey on *Brucella* infected animals like buffaloes [15, 33]. However, the high detection rates observed in buffaloes may be due to *B. abortus* being the common species in the ecosystem and is known to mostly infect bovine ungulates [33]. Generally, detection of zoonotic *Brucella* in wild animals in this study, especially *B. melitensis* and *B. abortus* that have already been found in the region, points to the possibility that they are the source of sustained *Brucella* transmission in livestock and humans in the interface areas of the Serengeti ecosystem. Transmission can be either from wild animals to livestock and vice versa, from wild animals to livestock then to humans or from wild animals directly to humans [17, 20]. Indeed it has been reported earlier, that wild animals can act as a source of infection for livestock and humans [34, 35].

Wildebeest migrate seasonally from the Serengeti to the Maasai Mara for pastures, a practice likely to spread *Brucella* in the Serengeti ecosystem [36]. Zebra constantly intermingle with wildebeest during grazing, living together in close association. This behavior could be the basis for the transmission of pathogens amongst wild animals [33, 37].

In this study, qPCR was observed to have a higher detection rate of *Brucella* spp. than AMOS PCR. This finding is supported by reports from other studies which compared the two platforms and reported qPCR as superior tool [29, 38, 39]. Most likely because the quantitative PCR is more sensitive to lower concentrations of DNA than conventional methods [29, 40]. It could however, also depend on the biotypes of *Brucella* circulating in the region, for example *B. abortus* biovar 3 which has previously been detected in Tanzania cannot be detected using the AMOS PCR [18, 41].

The AMOS PCR is designed to detect *B. abouts*, *B. melitensis*, *B. ovis* and *B. suis*, while the qPCR used in the present study was able to differentiate *B. abortus* and *B. melitensis* [29, 30, 42]. A positive result was considered only if a sample was positive by both assays. *B. suis* was detected in the AMOS PCR but could not be confirmed by the qPCR assay used. Future studies could build on these findings to conduct further molecular studies in wildlife samples, using more advanced typing techniques like the Bruce ladder [43] or multi-locus sequence analysis [41, 44].

This study had a number of limitations; first, the samples used in this study were collected on a convenience/availability basis and the study was not systematically designed to determine epidemiological inference for respective animal species. Secondly, although the qPCR assays sensitively detected *Brucella* DNA in these archived samples, we did not have sufficient quantities and quality of genomic DNA to confirm the species and sub-types using more advanced typing techniques. Lastly, although the study exploited molecular techniques on DNA extracted directly from clinical samples, archived for up to 15 years, most of the source material was of inadequate quality to perform serological testing or confirmatory culture. Future studies could target more freshly collected samples and explore options for immunological and bacteriology confirmatory tests in this critical yet under-studied population.

## Conclusions

Findings from this study show the robust use of molecular techniques for the detection of *Brucella* in DNA extracted directly from archived wild animal field samples. This has great potential in expanding the detection of brucellosis among populations that may be hard to reach or sample, and particularly in wild animals, where sample collection is expensive, dangerous and tedious. Numerous wildlife management and research institutions however, have samples in archive from previous field activities. This study has shown that there are *Brucella* spp. circulating in different wild animal species in the Serengeti ecosystem. Most of *Brucella* spp. detected have zoonotic potential. Detection of zoonotic *Brucella* species in wild animals suggests that livestock and humans at the interface areas are at risk of acquiring the infection, underscoring the need for a One Health approach for the control of this disease. The findings from this study, although contextual to the Serengeti ecosystem, provide valuable insights into *Brucella* infection and host associations in wild animal population applicable to much of sub-Saharan Africa.

## Supplementary Information

**Additional file 1: Supplementary materials S1.***Brucella* spp. detected by AMOS PCR from wildlife in the Serengeti ecosystem. The first and the last lanes are 10kb DNA ladder, lanes 2- 12, are positive samples, lanes 13 and 14 are negative samples, lane NC is a negative control containing nuclease free water and lane PC is a positive control comprising DNA of *B. abortus* strain RB51

**Additional file 2: Supplementary materials S2.** A data set with results from the three PCR assays (AMOS PCR, *Brucella* genus specific qPCR and qPCR speciation) performed.

## Data Availability

All data generated or analyzed during this study are included in this published article and its supplementary information files.

## Abbreviations

AMOS PCR: Multiplex polymerase chain reaction for *Brucella abortus*, *Brucella melitensis*,*Brucella ovis* and *Brucella suis*
DNA: Dioxyribonucleic acid
EDTA: Ethylenediaminetetraacetic acid
Kb: Kilobyte
PCR: Polymerase chain reaction
qPCR: Quantitative Real-Time PCR
SUA: Sokoine University of Agriculture
TAWIRI: Tanzania Wildlife Research Institute
WHO: World Health Organization
