# Supplementary figures and images for: Circulating Brucella species in wild animals of the Serengeti ecosystem, Tanzania
Source: One Health Outlook. 2021 Aug 24;3:15. doi: 10.1186/s42522-021-00047-6 (PMC8383352; doi:10.1186/s42522-021-00047-6)

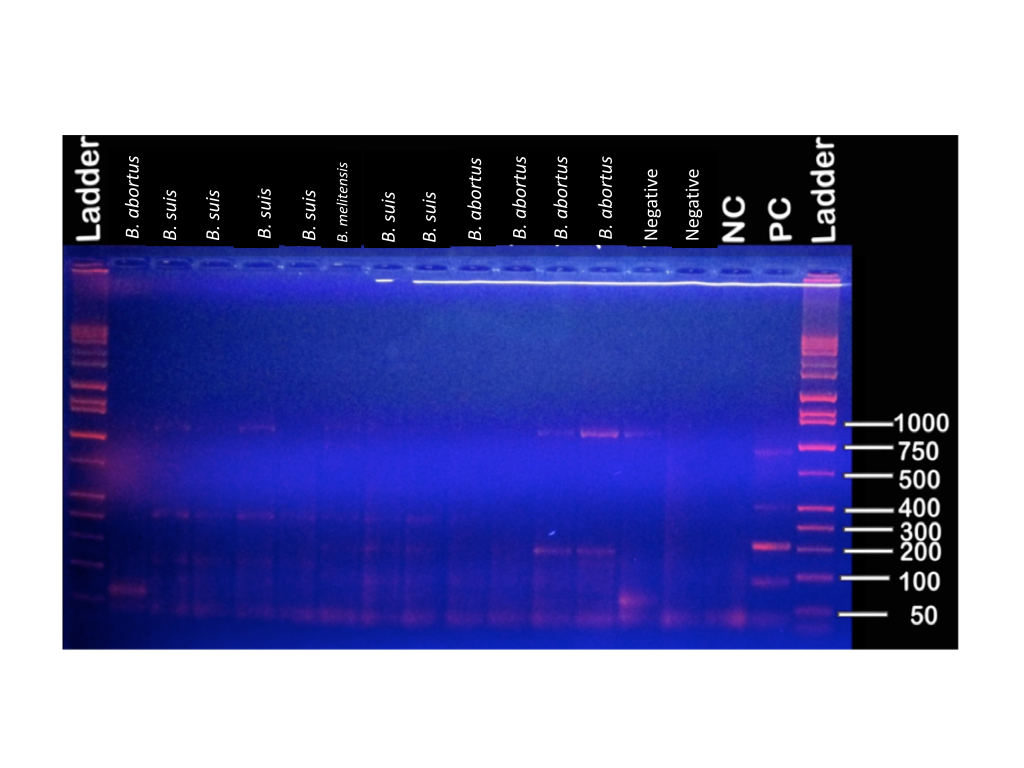

Supplement: Supplementary file 1 — Additional file 1: Supplementary materials S1.Brucella spp. detected by AMOS PCR from wildlife in the Serengeti ecosystem. The first and the last lanes are 10kb DNA ladder, lanes 2- 12, are positive samples, lanes 13 and 14 are negative samples, lane NC is a negative control containing nuclease free water and lane PC is a positive control comprising DNA of B. abortus strain RB51 [file 42522_2021_47_MOESM1_ESM.jpeg]
